# Main Bronchus Penetration by Thoracostomy Tube: A Rare Inadvertent Complication

**DOI:** 10.30476/BEAT.2020.85839

**Published:** 2021-01

**Authors:** Parviz Mardani, Reza Shahriarirad, Amirhossein Erfani, Keivan Ranjbar, Bizhan Ziaian, Armin Amirian, Hamed Ghoddusi Johari

**Affiliations:** 1 *Thoracic and Vascular Research Center, Shiraz University of Medical Science, Shiraz, Iran*; 2 *Department of Surgery, Shiraz University of Medical Sciences, Shiraz, Iran*; 3 *Student Research Committee, Shiraz University of Medical Sciences, Shiraz, Iran*

**Keywords:** Thoracostomy tube, Chest tube, Main bronchus, Misplacement, Bronchial injury

## Abstract

Tube thoracostomy has been known to be a common and invasive, however not innocuous, procedure which is often life-saving. Though, numerous complications have been reported during executing this procedure. In this report, we describe a 27-year-old woman, case of multiple trauma due to car collision that was transferred to our service due to severe right side chest tube air leak and subcutaneous emphysema in which after proper evaluation, it was revealed that the chest tube crossed through the right pleural cavity and penetrated the bronchus intermedius. A literature search failed to identify a similar case. The misplacement was confirmed by fiber optic bronchoscopy and after surgical and intensive care management of the patient, she was discharged with an uneventful post-op course. This case noticeably determines that bearing in mind the extreme risks and the careful checks of the tube location are required, particularly in trauma patients, even in the absence of anatomical abnormalities.

## Introduction

Thoracostomy tube insertion is often done under emergent, unexpected, and less monitored circumstances in the emergency department. All these factors contribute to increased complications, such as infection, malposition of the tube (as the most common), and organ injury [1-3]. Laceration of the lung due to the insertion of an intra-parenchymal tube is among the most common organ injuries which results in complications such as bleeding, sustained air leaks, bronchopleural fistula, infection, or lung abscess development. However, hemorrhage is more commonly seen during the removal of the tube rather than the placement. Furthermore, consideration of abrupt thoracotomy for the restoration of the laceration should be considered in severe and continues bleeding [4, 5]. In this report, we aimed to present a rare but devastating complication of mal-positioning of the thoracostomy tube.

## Case Presentation

A 27-year-old woman was the only survivor of a family in a car collision who developed multiple fractures and a lowered level of consciousness (GCS: 7); she was intubated and, due to pneumothorax, bilateral chest tubes (Size 28) were inserted for her at a local hospital by the emergency staff. After 5 days, she was transferred to our center due to severe right side chest tube air leak and subcutaneous emphysema. Evaluation at our center revealed left-hand fracture, bronchial injury, brain contusion, lung contusion, and T1 to T5 fracture. Sonography of the chest showed mild pleural effusion at the right side of the thoracic cavity with subsegmental collapse. Brain CT, abdominal sonography, echocardiography, and lab data were unremarkable.

Fiber optic bronchoscopy (FOB) was done via endotracheal tube (ETT). Severely inflamed trachea and thick plug in the orifice of the right upper lobe and entrance of the right main bronchus were observed and suctioning of the right and left main bronchus was done. No gross tracheal injury or rupture was seen, probably due to the thick plaque. The patient tolerated the procedure well and was transferred to surgery ICU.

**Fig. 1:**
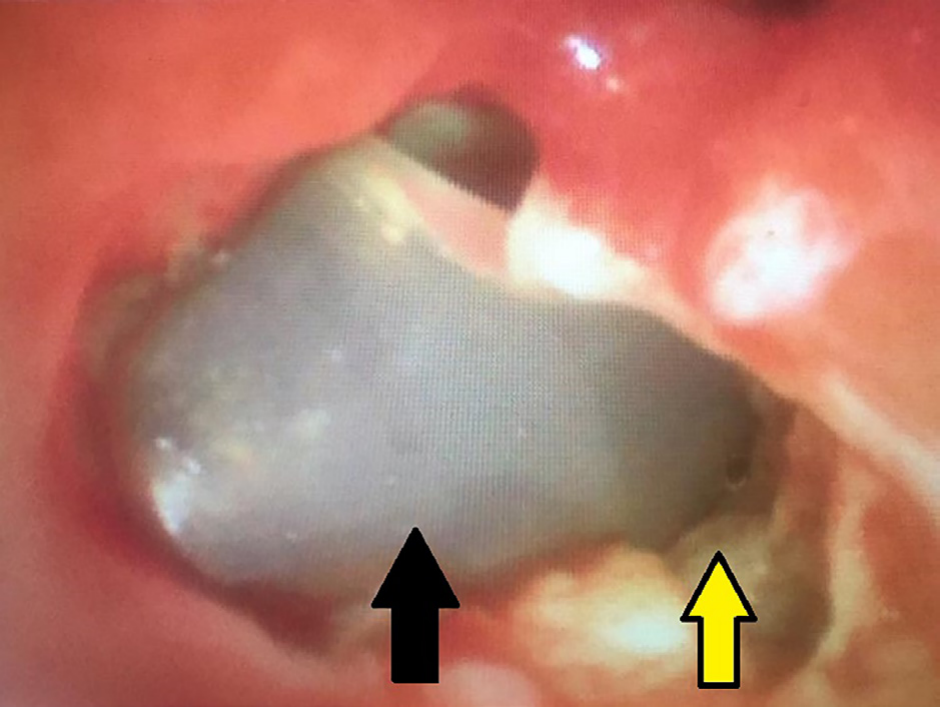
Fiber optic bronchoscopy revealing chest tube penetrating the bronchus intermedius; Yellow arrow demonstrating the point of entry of the chest tube into the right bronchus intermedius, at the origin of the middle and lower right lobe. (Black arrow)

Due to sustained air leakage, anther FOB was performed, and after pulmonary toilet and suction of all secretions and mucus, penetration of the chest tube was seen in the bronchus intermedius (Figure 1).

**Fig. 2:**
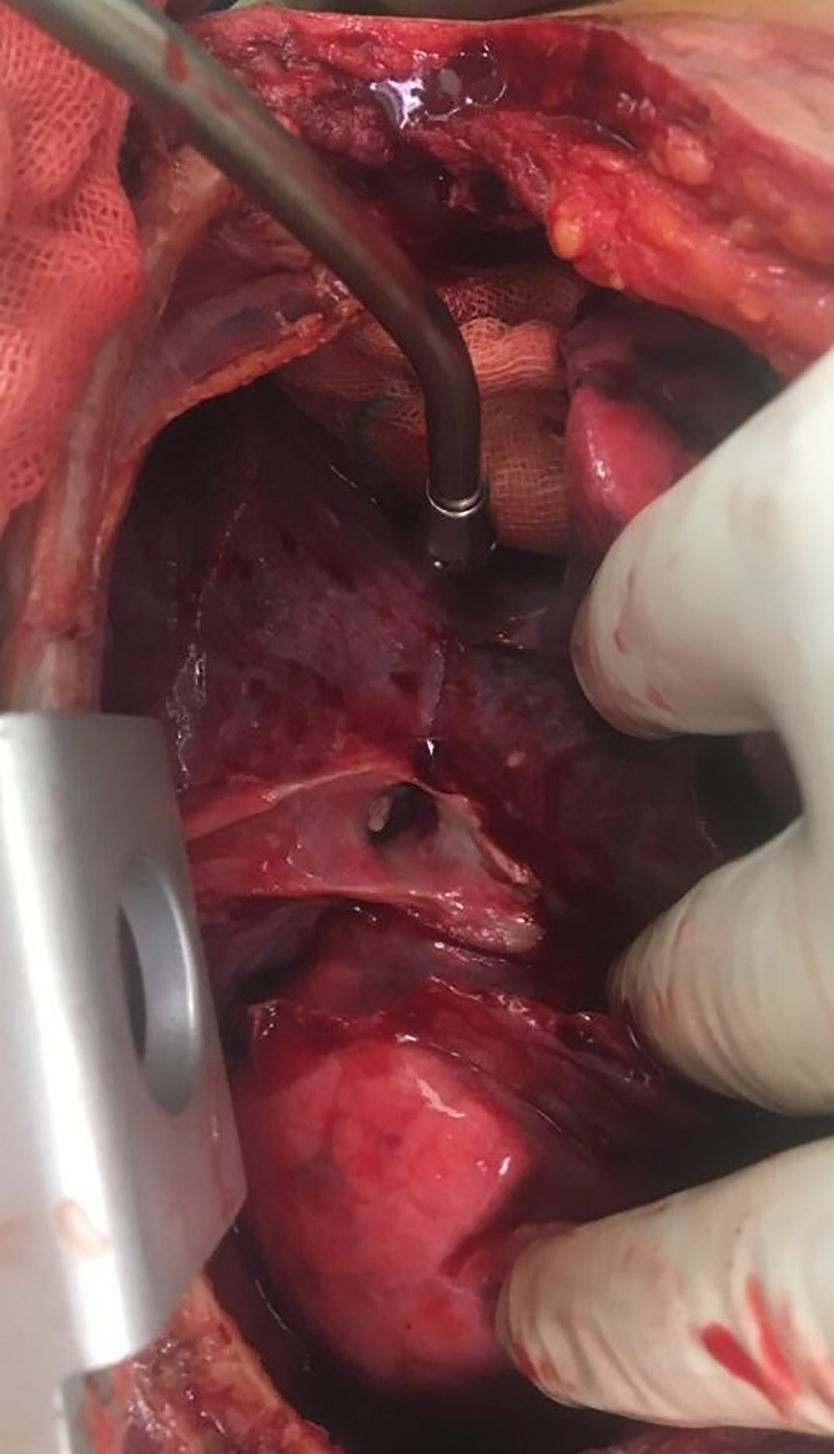
Chest tube penetrating the major fissure of the right side of the original middle lobe bronchus

**Fig. 3:**
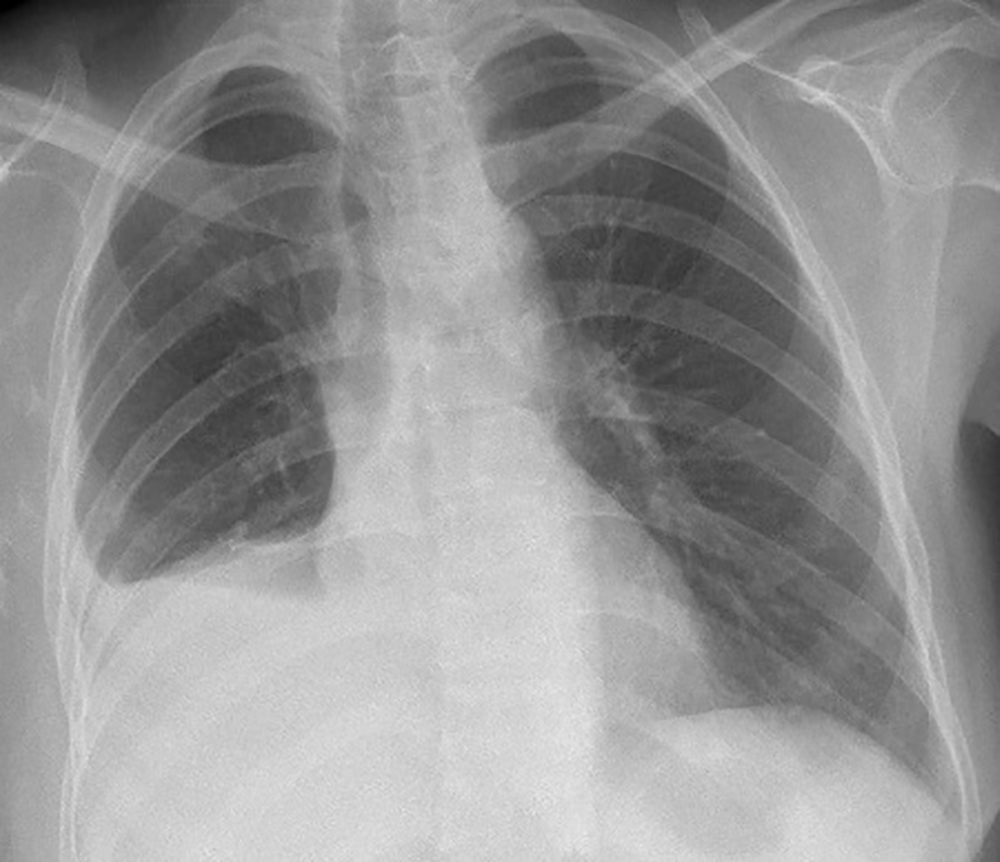
Post bilobectomy chest x-ray

Due to the penetration of the bronchus intermedius and massive air leak, the patient was scheduled for thoracotomy. The patient was transferred to OR and left double lumen tracheal intubation was done. After prep and drep, under general anesthesia, in the left decubitus position via a right posterolateral incision of the skin, the subcutaneous and intercostal muscles were opened and the chest cavity was entered. Pneumolysis was done and after releasing the inferior pulmonary ligament, the course of the right chest tube was followed in the major fissure which penetrated the bronchus intermedius at the origin of the middle and lower lobe (Figure 2). Chest tubes were removed. After evaluation and attempt for converting the patient to one lung, due to mal-positioning of the left double lumen, an anesthesiologist attempted to change the double lumen; during this change, the patient developed cardiac arrest that after resuscitation and change of the tube, the sinus tachycardia was obtained and the condition was acceptable. Based on the observation of penetration of the chest tube in the bronchus intermedius, we decided to perform bilobectomy for the patient. The procedure continued, and the inferior pulmonary vein was double ligated and cut, followed by the middle lobe pulmonary vein. Also, all branches of the middle and lower lobes of the pulmonary artery were double ligated and cut. Consequently, after transection of the proximal site of the bronchus intermedius injury, and stapling of the minor fissure with stapler No 8, bilobectomy was performed, followed by the closure of the stamp of the bronchus with Vicryl 3-0, and subsequently air leak test was done. Hemostasis was done and the chest cavity was irrigated with warm normal saline and saline betadine. Two chest tubes were inserted and then the chest was closed as routine. Afterwards, a tracheostomy tube (No. 8) was inserted through a longitudinal tracheal incision between the 2^nd^ and 4^th^ rings; after cuff inflation and normal capnography, FOB was done and all thick secretions were removed and the skin was closed with nylon 3-0 along with fixation of the tracheostomy tube to the skin with a nylon 3-0. The patient was observed and after she was stable, she was transferred to the ward; her condition improved and after 67 days of hospitalization and unremarkable post-op chest X-ray (Figure 3), she was discharged with no further complications.

## Discussion

Thoracostomy tube insertion is considered an invasive procedure done by emergency medicine physicians or trained personnel that places the patients at risk of complications such as empyema, misplacement of the tube, laceration of thoracic vessels, and injury to the heart, liver, spleen and lungs [2].

In our case, we had a very rare incident and due to the presence of pneumothorax, insertion of the thoracostomy tube was carried out that resulted in subcutaneous emphysema attributed to the penetration of the right main bronchus at the bronchus intermedius origin of the right middle lobe. There are few reports about the malposition of the thoracostomy tube in the literature. 

In 2007, Kerger *et al*. reported a 72-year-old patient with severe dyspnea and respiratory distress; the thoracostomy tube insertion in the right side resulted in positioning of the tube in the left atrium [6]. Kinjal Sethuraman et al. reported a 42-year-old man; following falling on spikes, he developed flank puncture wound and pleural effusion. After inserting the thoracostomy tube, it crossed through the retroperitoneal area and perforated the diaphragm [7]. Atikun Limsukon and colleagues also reported an 86-year-old woman with sepsis and massive right-side pleural effusion who developed a chylous effusion after a traumatic chest tube insertion into the lymphatic duct [8].

Rapidly performing thoracostomy tube insertion in emergent situations often results in the misplacement and complications, especially when done by a unexperienced physician. A study done by Kong in a high-volume trauma service demonstrated that junior doctors had a statistically significant higher complication (24%) rates while performing chest tube insertion compared to senior doctors with a complication rate of 5% (*p*<0.001) [9]. 

Chest tube insertion should be done in a so-called safe triangle with the anterior wall of the latissimus dorsi muscle and the horizontal line above the nipple level and pectoralis major muscle as the lateral border. Kong also stated that most complications occurred during the chest tube insertion outside the safety triangle (41%) [9]. A survey done by Griffiths and Roberts on 55 junior doctors showed that 45% of them were unaware of the correct anatomical location for chest tube insertion [10].

Evaluating the position of the thoracostomy tube after its insertion is of major importance, since the stiff end of a large-caliber drain can simply erode any structure that it is forced against. 

In conclusion, this case noticeably revealed that the extreme risks should be considered and the tube location should be carefully checked, particularly in trauma patients, even in the absence of anatomical abnormalities. Therefore, for minimizing the complications and preventing life-threating situations, performing chest tube insertion, especially in emergent situations, should be done by an experienced or senior physician while determining the correct depth of insertion, usually between 5 to 15cm, ensuring all ports are within the pleural cavity. We also recommend unforceful insertion of the chest tube while maintaining and cranial positioning of the tip of the tube when entering the chest wall, following the standard techniques. 

## References

[1] Ball CG, Lord J, Laupland KB, Gmora S, Mulloy RH, Ng AK (2007). Chest tube complications: how well are we training our residents?. Can J Surg..

[2] Al Mosa AFH, Ishaq M, Ahmed MHM (2018). Unusual Malposition of a Chest Tube, Intrathoracic but Extrapleural. Case Rep Radiol..

[3] Santos C, Gupta S, Baraket M, Collett PJ, Xuan W, Williamson JP (2019). Outcomes of an initiative to improve inpatient safety of small bore thoracostomy tube insertion. Intern Med J..

[4] Kwiatt M, Tarbox A, Seamon MJ, Swaroop M, Cipolla J, Allen C (2014). Thoracostomy tubes: A comprehensive review of complications and related topics. Int J Crit Illn Inj Sci..

[5] Filosso PL, Guerrera F, Sandri A, Roffinella M, Solidoro P, Ruffini E (2017). Errors and Complications in Chest Tube Placement. Thorac Surg Clin..

[6] Kerger H, Blaettner T, Froehlich C, Ernst J, Frietsch T, Isselhorst C (2007). Perforation of the left atrium by a chest tube in a patient with cardiomegaly: management of a rare, but life-threatening complication. Resuscitation..

[7] Sethuraman KN, Duong D, Mehta S, Director T, Crawford D, St George J (2011). Complications of tube thoracostomy placement in the emergency department. J Emerg Med..

[8] Limsukon A, Yick D, Kamangar N (2011). Chylothorax: a rare complication of tube thoracostomy. J Emerg Med..

[9] Kong VY, Oosthuizen GV, Sartorius B, Keene C, Clarke DL (2014). An audit of the complications of intercostal chest drain insertion in a high volume trauma service in South Africa. Ann R Coll Surg Engl..

[10] Griffiths JR, Roberts N (2005). Do junior doctors know where to insert chest drains safely?. Postgrad Med J..

